# Matrine Combined with Mammalian Target of Rapamycin Inhibitor Enhances Anti-Tumor Efficacy of dendritic cell Vaccines in hepatocellular carcinoma

**DOI:** 10.1080/21655979.2022.2037855

**Published:** 2022-04-10

**Authors:** Ning Zhou, Sheng Li, Fan Zhang, Cong Chen, Yumin Li

**Affiliations:** a Department of Gastroenterology, The First People’s Hospital of Lanzhou City, Lanzhou Gansu, China; b Key Laboratory of Digestive System Tumors of Gansu Province, Lanzhou Gansu, China

**Keywords:** Matrine, mTOR inhibitor, dendritic cells, Huh7 cells, CTL, vaccine, nude mice

## Abstract

Dendritic cells (DCs), as the most important antigen-presenting cells, play a crucial role in T cell activation. The latest research showed that inhibition of the mammalian target of rapamycin (mTOR) could enhance DCs maturation, promoting antigen presentation. Matrine has been identified as one of the key alkaloids isolated from the roots of *Sophora flavescens*. In present study, we combined matrine and mTOR inhibitor KU0063794 to observe the DCs functions, especially the antigen presentation ability. DCs were activated by phosphate-buffered saline (PBS), lipopolysaccharide (LPS), LPS+KU0063794, LPS+Matrine, and LPS+KU0063794+Matrine. The surface markers in DCs, proliferation of T cells and cytokines were detected by flow cytometry, cell counting kit-8 (CCK-8) and enzyme-linked immunosorbent assay (ELISA), respectively. The lactate dehydrogenase (LDH) release test was used to detect the antitumor efficacy. The tumor growth curves were plotted by calculating tumor volume. The apoptosis was detected by Terminal-deoxynucleoitidyl Transferase-Mediated Nick End Labeling (TUNEL) method. Matrine combined with KU0063794 could enhance the maturity of DCs, T cells proliferation and cytokines secretion (*P* < 0.05). The cytotoxic T lymphocytes (CTL) killing efficacy of LPS+KU0063794+Matrine group was higher than other groups *(P *< 0.05). *In vivo*, the tumor weights and volumes in LPS+KU0063794+Matrine group were lower than other groups. The detections of tumor apoptosis were increased in LPS+KU0063794+Matrine group (*P* < 0.05). DC vaccine with mTOR inhibitor and matrine could significantly improve the efficacy of antitumor immunity *in vitro* and vivo. These findings illustrated that mTOR inhibitor and matrine, as two immunomodulators, could enhance DC activation and differentiation.

## Graphical Abstract

**Figure:**
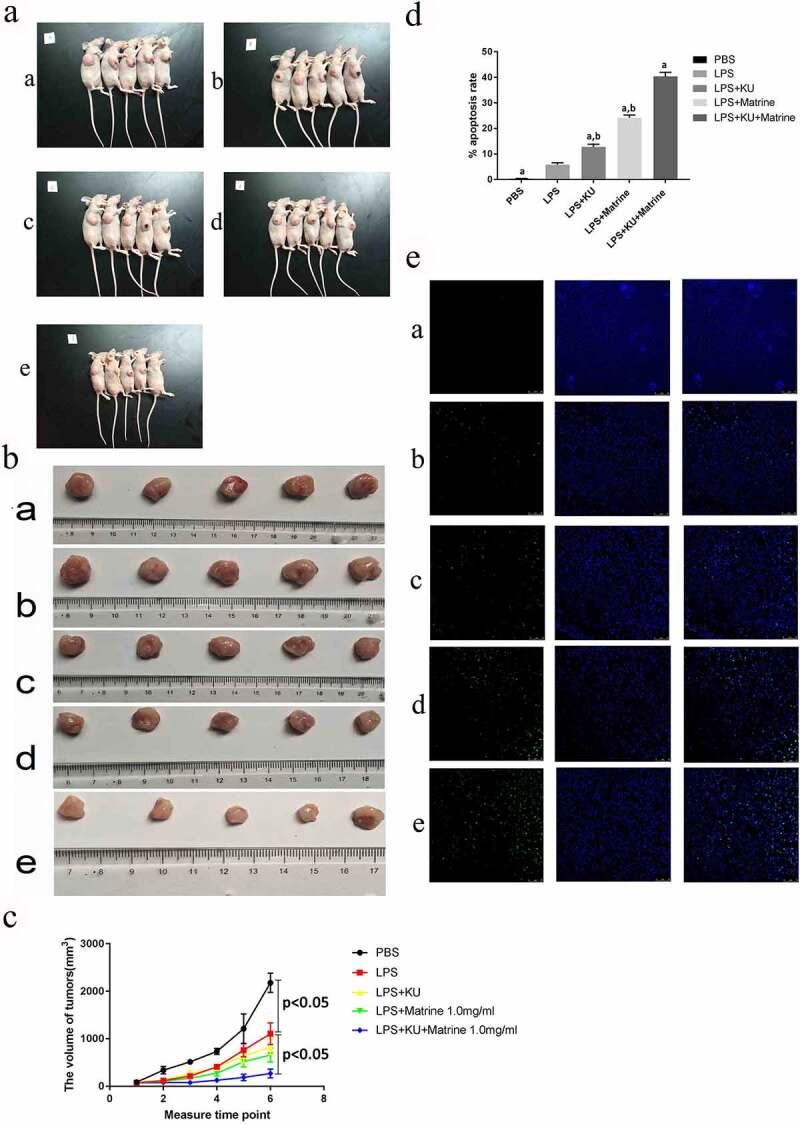

## 1. Introduction

Dendritic cells (DCs) are key antigen-presenting cells, which could capture, process and present the ‘foreign’ antigen to naive T cells. It is desired to improve the ability of antigen presentation in DCs for cancer immunotherapy [1]. The mammalian target of rapamycin (mTOR) network is important for numerous cellular processes. Several studies showed that mTOR signaling pathways took part in regulating DCs functions [2]. The inhibition of mTOR in mature DCs (mDCs) could enhance the antigen presentation ability. Moreover, traditional Chinese medicine (TCM) also plays an important role in regulating immune responses [3–5]. Matrine is a kind of main alkaloid components, which were isolated from the classic TCM *Sophora* roots [6–9]. It has been recently reported that matrine is associated with activation of autophagy, apoptosis and numerous immune related signal pathways. To the best of our knowledge, our study was the first study to investigate the combination of matrine and mTOR inhibitor KU0063794 to improve the antigen presentation ability of DCs and the antitumor efficacy of DC vaccine for HCC patients. The aim and goals of this study are to combine matrine and mTOR inhibitor KU0063794 to observe the DCs’ functions, especially the antigen presentation ability. Our hypothesis is that DC vaccine with mTOR inhibitor and matrine could significantly improve the efficacy of antitumor immunity *in vitro* and vivo. Compared to previous works by other researchers, our results indicated that the combination of matrine and KU could promote DC maturation and enhance T cell proliferation

## 2. Materials and methods

### 2.1 Preparation for dendritic cells and T cells

Peripheral blood mononuclear cells were isolated from blood samples of healthy donors by using lymphocytes as a separating solution (Solarbio, Beijing, China). Adherent cells were used to incubate and differentiate DCs. MD-STM-N medium (MD Pacific, Tianjing, China) and 10% fetal bovine serum (FBS) (GIBCO, Thermo Fisher Scientific, Inc., Waltham, MA, USA) were used for cell culture. Meanwhile, differentiation of DCs was added to essential cytokines, such as GM-CSF (10 μg/ml, PeproTech, Annoron, Beijing, China) and IL-4 (10 μg/ml, PeproTech, Annoron, Beijing, China). The non-adherent cells were collected for T cells incubation. IL-2 (50 U/ml, PeproTech, Annoron, Beijing, China) and IL-7 (10 ng/ml, PeproTech, Annoron, Beijing, China) were added to induce proliferation of T cells. The system used for densitometry is Human Lymphocyte Separation Solution (Tianjin Haoyang Biological Products Technology Co., LTD.)

### 2.2 Preparation for liver tumor antigen and loaded on DCs

The Huh7-associated antigens were generated by thawed and refreezed circling. Then, they were added into the immature DC on the fifth day of culturation. The ratio between DCs and tumor antigen was 1:20. The imDCs were identified during the incubation on the fifth day. After loading the Huh7-associated antigens within 48 hours, the DCs were incubated as semimature DC (semiDC). The mDCs were harvested after 24 hours interventions by KU0063794 and/or matrine [5].

### 2.3 Pharmacological intervention

After 48 hours of semiDC incubation, the DCs were randomly divided into five groups: A, B, C, D and E (Group A: Untreated group, Group B: LPS, Group C: LPS+KU0063794, Group D: LPS+Matrine, Group E: LPS+KU0063794+Matrine. The untreated group was added 1 mL phosphate-buffered saline (PBS) only. The four DC groups were stimulated with 100 ng/mL LPS in the absence or presence of KU0063794 (100 nM) and/or matrine (1 mg/ml), respectively. The five groups were cultured in MD-STM-N medium at 37°C in a humidified incubator supplemented with 50 mL/L CO_2_ [6].

### 2.4 *Induction of CTL cells and detection of CTL killing effects* in vitro

Five DC groups described in part 2.3 were mixed with T cells for 7 days at a ratio of 1:10, cocultured in RPMI medium 1640 containing 10% FCS, 50 U/ml IL-2 (PeproTech, Annoron, Beijing, China) and 10 ng/ml IL-7 (PeproTech, Annoron, Beijing, China). Every 3 days, a new culture solution (at half volume) was replaced. On day 7, the activated CTL cells were collected for killing effect tests as the effector cells. Huh7 cells as the target cells, the ratios of the effector cells to the target cells were 1:1, 5:1, 10:1, 50:1, 100:1, respectively. Co-cultured for 5 hours, then the supernatant was collected CTL killing effects by using LDH Kit (Promega corporation, Madison, USA) [7].

### 2.5 Detection of biomarkers of DCs by flow cytometry

The maturation of DCs was detected by flow cytometry after 24 hours pharmacological intervention by KU0063794 and matrine, using CD83 antibody, CD86 antibody and HLA-DR antibody (BD Biosciences, Franklin Lakes, NJ, USA) [8].

### 2.6 Proliferation of T cells

Proliferation of T cells was detected by CCK-8 method. The DCs in each group were disposed with different pharmacological intervention mixed with T cells. A CCK-8 test kit (Dojindo, Shanghai, China) was used, and a specific operation was conducted according to the manufacturer’s instructions [9].

### 2.7 Cytokine measurement

When DCs in each group were disposed with different pharmacological interventions mixed with T cells, the supernatants were used to detect for IFN-γ, TNF-α and IL-10 by ELISA method (NeoBioscience company, Shenzhen, China) [10].

*Study on mTOR inhibitor-induced maturation of human peripheral blood monocyte-derived DCs.*

The monocytes were isolated from the peripheral blood of healthy volunteers by density gradient centrifugation method. The monocytes were induced to differentiate into DCs under the action of GM-CSF and IL-4 by adherent culture method. The DCs were sensitized with Huh7 cell lysates. Group B: Lipopolysaccharide (LPS); Group C: LPS+KU0063794 (mTOR inhibitor); Group D: LPS+ Matrine; Group E: LPS+KU0063794+Matrine stimulated DC maturation, and finally prepared as liver cancer DC vaccine in each group. PBS stimulated group (group A) was used as blank control group. The expression of surface markers CD83, HLA-DR and CD86 were detected by flow cytometry at different culture time points.

Experimental study on T cell proliferation induced by mTOR inhibitor. The prepared HEPATocellular carcinoma DC vaccines were co-cultured with the initial T cells in a ratio of 1:10 for 72 h, and the PROLIFERATION of T cells was detected by CCK-8 method.

*Study on the ability of mTOR inhibitor to induce CTL cells to secrete cytokines.*

Hepatocellular carcinoma DC vaccine in each group was co-cultured with primary T cells to activate the primary T cells into cytotoxic T lymphocytes (CTL). The secretion of IFN-γ, TNF-α and IL-10 at different time points during CTL induction was detected by ELISA.

*Study on specific killing efficiency of CTL to hepatocellular carcinoma cells in vitro and in vivo.*

CTL cells were used as effector cells and Huh7 cells as target cells after 21 days of induction for 5 h in the ratios of 1:1, 5:1, 10:1, 50:1 and 100:1, respectively. LDH release assay was used to detect in vitro specific killing efficacy of hepatocellular carcinoma DC vaccine induced by LPS and LPS+mTOR inhibitor.

Hepatocellular carcinoma huH7-bearing nude mice model was established and randomly divided into group A: blank control group. Group B: LPS-MDC-CTL; Group C: LPS+ KU0063794-MDC-CTL group; Group D was LPS+MATRine-MDC-CTL. Group E: LPS+KU0063794+MATRine-MDC-CTL group was used for immunotherapy, once a week, 3 times in total. Tumor volume and weight were measured, tumor growth curve was drawn, and apoptosis was detected by TUNEL method.

### 2.8 Tumor challenge experiments

The nude mice were purchased from Slake Laboratory Animal Company (Shanghai, China) and subcutaneously inoculated with 2 × 10^7^ human Huh7 cells under the right forelimb subaxillary. When the tumor grew to 5–7 mm (about 80–100 mm^3^), the mice were randomly divided into five groups: A, B, C, D and E (Group A: blank control group, Group B: LPS-mDC-CTL. Group C: LPS+KU0063794-mDC-CTL group, Group D: LPS+Matrine-mDC-CTL group; Group E: LPS+KU0063794+Matrine-mDC-CTL group). The mice were injected with 0.2 ml PBS in Group A (blank control group). For the four intervention groups, they were injected with human CTLs described in part 2.4, respectively. The doses of autologous CTLs were 2 × 10^7^ in each group. The intervention was performed once a week, 3 times in total. Mice were monitored for tumor growth periodically, and tumor sizes were measured with digital calipers (Fisher Scientific). The tumor tissues were harvested after 7 days for the last immunization. The mice were sacrificed and the tumor tissues with final tumor volumes and weights were measured. Apoptosis of tumor tissues was detected by TUNEL (Roche, Switzerland) staining [11]. After resuscitation, the CONCENTRATION of CTL cells was adjusted to 108/ml, and the nude mice with tumor size of 5–7 mm were selected and successfully inoculated for 3 weeks, once a week for 3 times in total. Group A (blank control group) was injected with 0.2 ml PBS, and GROUP B–E was hepatocellular carcinoma – DC vaccine prepared after different interventions stimulated DC maturity, and CTL cells prepared by 2.4 steps: group B–E was injected with 0.2 ml CTL cells (2 × 10^7^ cells/mouse). The treatment was peritumoral multipoint injection.

### 2.9 Statistical analysis

Data were presented as percentages, means with standard deviation (mean ± SD). One-way analysis of variance (ANOVA) was used for the comparison of mean values among 5 groups. LSD test was used for comparison between groups. *P* < 0.05 was considered statistically significant. All statistical analyses were performed using SPSS23.0 for Windows (SPSS, Chicago, IL, USA).

## 3 Results

In the present study, we combined matrine and mTOR inhibitor KU0063794 to observe the DCs functions, especially the antigen presentation ability. The activations of DC were stimulated by PBS, LPS, LPS+KU0063794, LPS+Matrine, and LPS+KU0063794+Matrine. The surface markers in DCs, proliferation of T cells and cytokines were detected by flow cytometry, CCK-8 and ELISA, respectively. The LDH release test was used to detect the antitumor efficacy. The tumor growth curves were plotted by calculating tumor volume. The apoptosis was detected by the TUNEL method. The results showed that matrine combined with KU0063794 could enhance the maturity of DCs, T cells proliferation and cytokines secretion (*P* < 0.05), compared with other groups. The CTL killing efficacy of LPS+KU0063794+Matrine group was higher than other groups *(P *< 0.05). *In vivo*, the tumor weights and volumes in LPS+KU0063794+Matrine group were lower than other groups. The detections of tumor apoptosis were increased in LPS+KU0063794+Matrine group than other groups (*P* < 0.05). DC vaccine with mTOR inhibitor and matrine could significantly improve the efficacy of antitumor immunity *in vitro* and vivo. These findings illustrated that mTOR inhibitor and matrine, as two immunomodulators, could enhance DC activation and differentiation.

### 3.1 Matrine combined with mTOR inhibitor KU0063794 could enhance the maturity phenotypes of DCs

**Figure 1.**
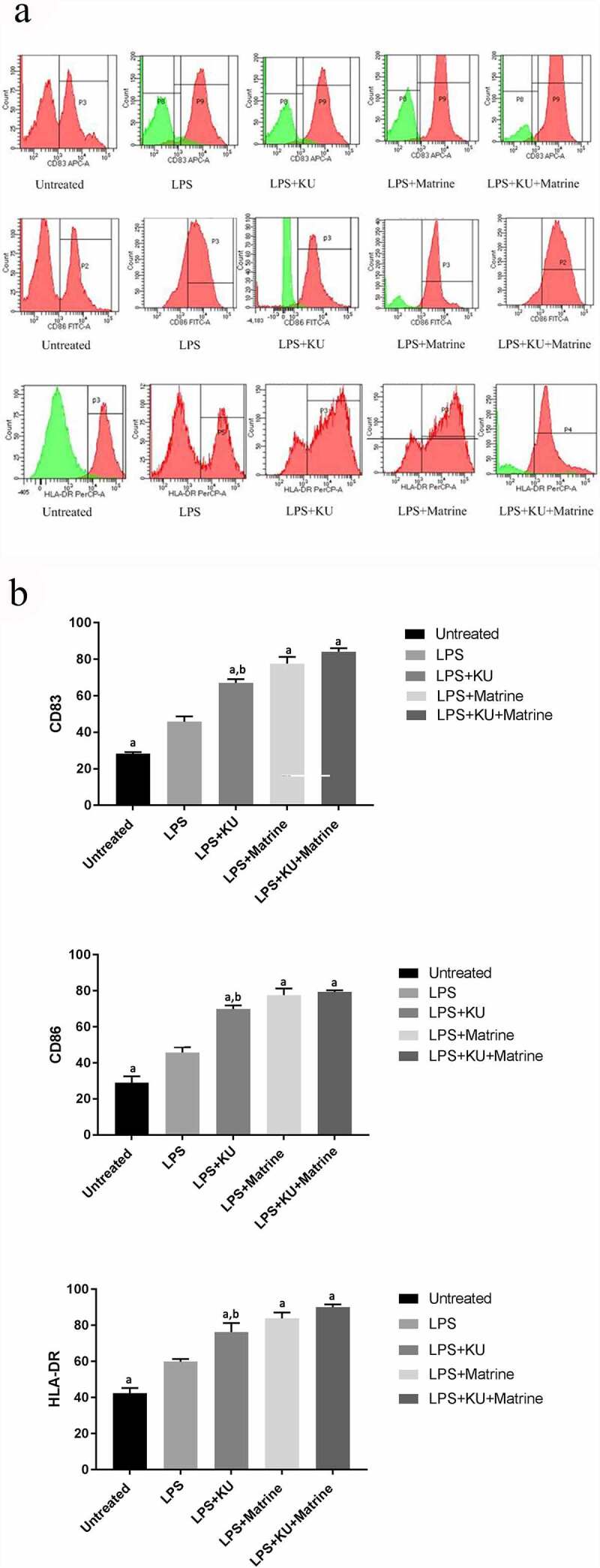
Matrine combined with KU0063794 could enhance the maturity phenotype of DCs.

After pharmacological intervention with KU0063794 and/or matrine, the expression of CD83, CD86 and HLA-DR of DCs were increased. The expression of CD83, CD86 and HLA-DR in KU0063794 combined with matrine group showed the highest level among all intervention groups (*P* < 0.05). These results suggested that matrine or KU0063794 could promote DCs maturation, and the combined effect of the two drugs was greater (Figure 1).

(A) The expressions of CD83, CD86 and HLA-DR on mDCs tested by flow cytometry after pharmacological intervention for 24 h in different groups. (B) The comparisons of CD83, CD86 and HLA-DR expressions among five groups. ^a^*P<*0.05 vs. LPS group; ^b^*P*<0.05 vs. LPS+KU+Matrine group.

### 3.2 Matrine combined with mTOR inhibitor KU0063794 could potentiate the capacity of DCs to induce T-cell proliferation

**Figure 2.**
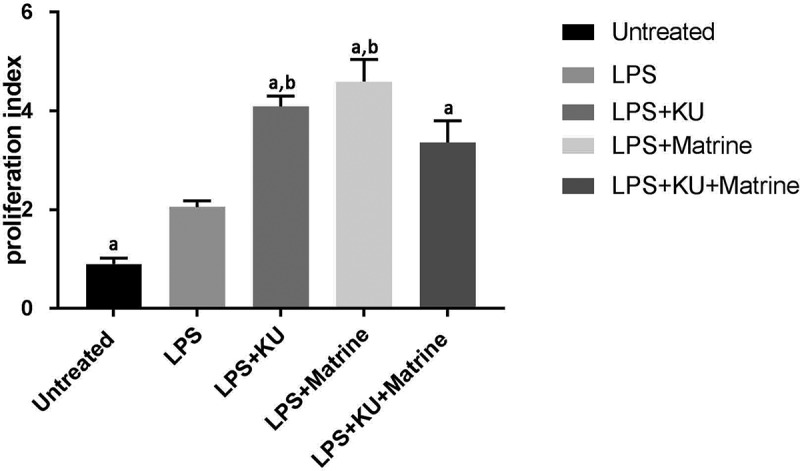
Proliferation index of T cells co-cultured with different groups of DCs was detected by CCK-8 method. ^a^*P<*0.05 vs. LPS group; ^b^*P*<0.05 vs. LPS+KU+Matrine group.

Compared with the LPS group, the efficacy of KU0063794 and matrine could significantly enhance the T cells’ proliferation (*P* < 0.05). And the combination of KU0063794 and matrine had the strongest effect (Figure 2).

### 3.3 *Matrine combined with mTOR inhibitor KU0063794 could induce CTLs to secrete IFN-γ and TNF-α, whereas suppress IL-10*

**Figure 3.**
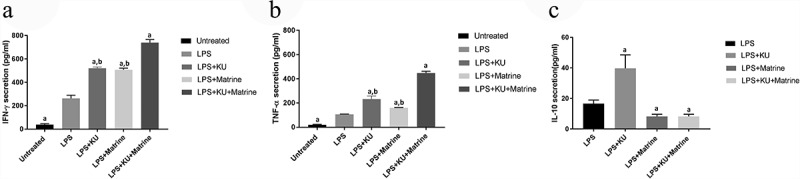
The HCC-DC vaccine induced by KU and matrine could influence the cytokine secreting ability of CTL cells.

IFN-γ and TNF-α in the mixed system were increased after pharmacological intervention with KU0063794 and/or matrine, the groups of combination drugs expressed more IFN-γ and TNF-α than the groups used a drug alone or no drug intervention. At the same time, for IL-10, results were on the contrary, combination drug groups expressed less IL-10 than the LPS group. But the LPS+KU0063794 group had higher IL-10 secretion than LPS group (*P < *0.05) (Figure 3).

(A) The expression of IFN-γ was detected by ELISA. ^a^*P<*0.05 vs. LPS group; ^b^*P*<0.05 vs. LPS+ KU+Matrine group. (B) The expression of TNF-α was detected by ELISA. ^a^*P<*0.05 vs. LPS group; ^b^*P*<0.05 vs. LPS+KU+Matrine group. (C) The expression of IL-10 was detected by ELISA. ^a^*P<*0.05 vs. LPS group.

### 3.4 *Matrine combined with mTOR inhibitor KU0063794 could heighten the CTL killing effect* in vitro

**Figure 4.**
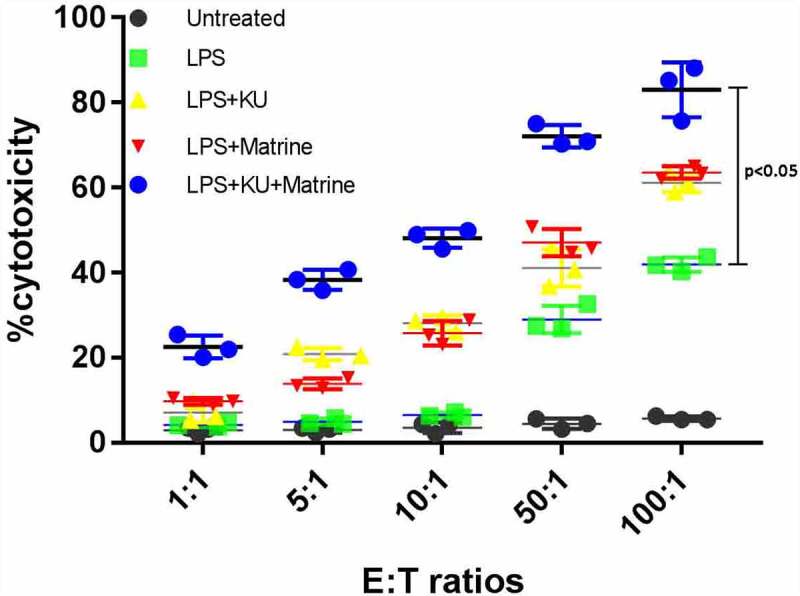
The specific killing effects of KU combined matrine induced HCC DC vaccine in vitro. The killing effects of DC vaccine increased with the increased E:T ratio.

The CytoTox-96 assay (Promega corporation, Madison, USA) evaluated cytotoxicity by assessing the total release of LDH into culture medium as a consequence of damaged cell membranes. LPS+KU, LPS+Marine, LPS+KU+Matrine groups expressed more LDH than untreated and LPS groups (*P* < 0.05). The LPS+KU+Matrine group was the best one, indicating that combination of matrine and KU0063794 on DCs induced stronger CTL killing effect than any drug alone or LPS group. At the same time, the killing effect increased with the increase of effector:target cell ratio (Figure 4).

### 3.5 *Matrine and KU enhanced the ability of HCC-DC vaccine therapeutic antitumor immunity* in vivo

**Figure 5.**
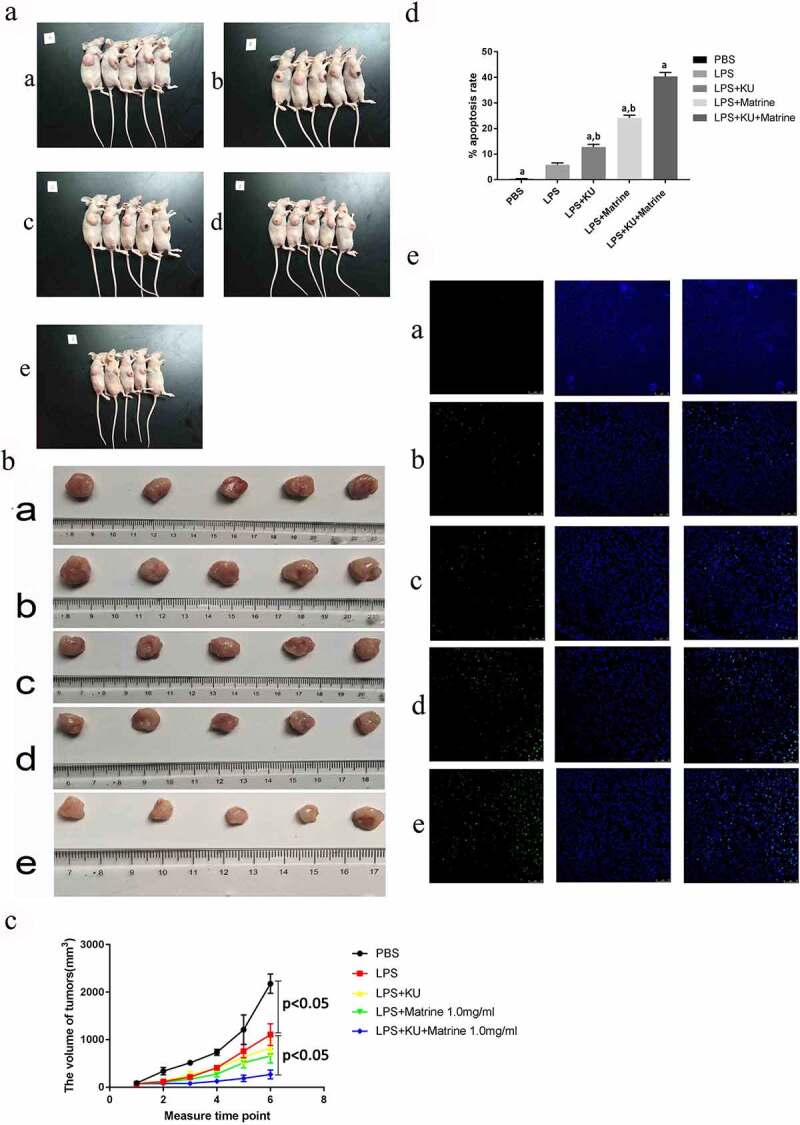
Matrine and KU enhanced the ability of HCC-DC vaccine therapeutic antitumor immunity in vivo.

After CTL immunotherapy, tumor weight and volume of nude mice in LPS+KU0063794+Matrine group were significantly lower than those in the LPS group, with significant tumor inhibition effect (*P* < 0.05) (Figures 5a–5c). Compared with the LPS group, the degree of tumor apoptosis in the LPS+mTOR inhibitor+matrine group was significantly increased (*P* < 0.05) (Figure 5d–5e).

(A) Images of xenograft nude mice models. (B) Transplantation tumor specimens of nude mouse. (C) Images of tumor growth curve. (D) The apoptosis rates in different groups (*P < 0.05). (E) TUNEL staining of the tumor apoptosis in different groups (×200).

## 4. Discussion

DC is a full-time antigen-presenting cells, which can effectively induce T cell immune response. The autologous DC vaccine plays a critical role in antitumor immunotherapy. However, the efficacy of DC vaccine is still unsatisfactory in clinical applications. It has been found that the efficacy of DC vaccine mostly relies on the degree of mature DCs. As is well known, mTOR is one of the serine–threonine kinases [10]. Its network plays an important role in integrating intra- and extracellular nutrient to control basic cellular processes, such as metabolism and protein synthesis [12]. In the present study, we found that inhibition of mTOR in incubation of mature DCs could enhance the presentation of endogenous self-antigens on MHCII molecules [11]. Traditional Chinese medicines (TCMs) are garnering increasing attention in immune therapy. Accumulating evidence indicated that some TCMs affect the functions of immune cells, such as DCs [13,14]. Jing et al. reported that astragalus polysaccharide could promote the maturity of DC cells and enhance the ability of antigen presentation [15]. Lin et al. reported that the treatment of DCs with ganoderma lucidum polysaccharides resulted in improving cell surface of CD80, CD86, CD83 expression; increasing the secretions of IL-12p70/p40; and enhancing T cell-stimulatory capacity [16]. Matrine (C_15_H_24_N_2_O) is one of the key tetracyclo-quinolizindine alkaloids isolated from the roots of *Sophora flavescens* Aiton [17]. The antitumor effect of matrine has been well documented, but the effects on DCs maturation and antigen presentation were not be reported before [18,19].

Our results indicated that the combination of matrine and KU could promote DC maturation and enhance T cell proliferation (*P* < 0.05). DC regulates cytokines to influence the development of different T helper cells [20]. With the development of research, the heterogeneity of DC has been identified, such as DC1, DC2 and DC17 [21]. DC1 induces polarization of Th1. Th1 cells secrete high levels of IFN-γ and TNF-α, which is associated with antitumor immunity. Th2 cells secrete high levels of IL-4 and IL-10. It is suggested that Th2 cytokine dominance is one of the mechanisms of tumor immune escape. From our study, the expression levels of IFN-γ and TNF-α were significantly increased by using KU0063794 and Matrine, meanwhile interleukin-10 (IL-10) was decreased (*P* < 0.05). Matrine combined with mTOR inhibitor can induce and stimulate the phenotype and functional maturity of DC, which is more likely to induce Th1 and/or Th17 type immune response. The mature DCs induced by induction may be more DC1 phenotype pedigree, providing a way to optimize the selection of DC phenotype in the preparation of DC vaccine. According to previous reports, mTOR inhibitors positively affected the production of proinflammatory cytokines such as TNF-α, and inhibited IL-10 production [22–24]. Activating mTOR could help strongly translate IL-10 Hence, inhibition of mTOR decreased production of IL-10 in DCs. However, in this study, we found that the LPS+KU group had significantly higher IL-10 secretion than LPS group [25–27]. mTOR plays an inhibitory role in regulating IL-10, which is different from the above studies, but the specific mechanism needs further research. After pharmacological intervention, LPS+KU, LPS+Matrine and LPS+KU+Matrine groups expressed more LDH than untreated and LPS groups. Moreover, compared with any single-drug group, the combined drug groups showed stronger killing power (*P* < 0.05). To further verify whether KU combined with matrine can enhance antitumor efficacy *in vivo*, we conducted animal experiments [28]. Both tumor volume and tumor weight in LPS+KU+Matrine group were smaller than those in LPS, LPS+KU0063794 and LPS+matrine groups. The apoptosis rates of tumor cells were significantly higher in LPS+ mTOR inhibitor+matrine than that in other groups, indicating that CTL induced by LPS+mTOR inhibitor +Matrine group had the strongest killing ability *in vivo*, which was consistent with the results *in vitro*.

The ability of antigen presentation of DCs was related to autophagy levels in activated DCs, more active of autophagy, more stronger of antigen presentation ability. Matrine could induce autophagy was well documented. According to our results, we speculated that matrine promoted antigen presentation of DCs might be related with autophagy [29–32]. mTOR inhibits autophagy through its recruitment into the Atg1/ULK1–mAtg13–FIP200 autophagy initiation complex and subsequent Ser757 phos – phorylation of ULK1 [33]. Therefore, the combination of matrine and mTOR inhibitor might further enhance autophagy, and then improve the ability of antigen presentation in DC cells. The significance of this study is that we found mTOR inhibitor and matrine could enhance DC activation and differentiation. LPS+mTOR inhibitor+Matrine could induce DC maturation in the preparation of DC vaccine. The combination of matrine and mTOR inhibitor might further enhance autophagy.

## 5. Conclusions

The combination of mTOR inhibitor and matine could enhance the efficacy of HCC-DC vaccine. It was suggested that LPS+mTOR inhibitor+Matrine could be used as a new mixture to induce DC maturation in the preparation of DC vaccine. The combination of matrine and mTOR inhibitor might further enhance autophagy.

## Highlights

- mTOR inhibitor and matrine could enhance DC activation and differentiation.
- LPS+mTOR inhibitor+Matrine could induce DC maturation in the preparation of DC vaccine.
- The combination of matrine and mTOR inhibitor might further enhance autophagy

## References

[1] Plumas J. Harnessing dendritic cells for innovative therapeutic cancer vaccines. Curr Opin Oncol. 2021 Dec 20. DOI:10.1097/CCO.0000000000000815 34930882

[2] Sukhbaatar N, Hengstschläger M, Weichhart T. mTOR-mediated regulation of dendritic cell differentiation and function. Trends Immunol. 2016;37(11):778–789. 27614799 10.1016/j.it.2016.08.009 PMC6095453

[3] How CW, Ong YS, Low SS, et al. How far have we explored fungi to fight cancer? Semin Cancer Biol. 2021 Mar 15;S1044-579X(21):00059–6. 10.1016/j.semcancer.2021.03.009 33737109

[4] Tan KL, Chia WC, How CW, et al. Benchtop isolation and characterisation of small extracellular vesicles from human mesenchymal stem cells. Mol Biotechnol. 2021 Sep;63(9):780–791. 34061307 10.1007/s12033-021-00339-2

[5] Sharma MD, Pacholczyk R, Shi H, et al. Inhibition of the BTK-IDO-mTOR axis promotes differentiation of monocyte-lineage dendritic cells and enhances anti-tumor T cell immunity. Immunity. 2021 Oct 12;54(10):2354–2371. 34614413 10.1016/j.immuni.2021.09.005 PMC8516719

[6] Looi QH, Foo JB, Lim MT, et al. How far have we reached in development of effective influenza vaccine? Int Rev Immunol. 2018;37(5):266–276. 30252547 10.1080/08830185.2018.1500570

[7] Huang H, Du T, Xu G, et al. Matrine suppresses invasion of castration-resistant prostate cancer cells by downregulating MMP-2/9 via NF-κB signaling pathway. Int J Oncol. 2017;50(2):640–648. 28000853 10.3892/ijo.2016.3805 PMC6903897

[8] Gu YY, Chen MH, May BH, et al. Matrine induces apoptosis in multiple colorectal cancer cell lines in vitro and inhibits tumour growth with minimum side effects in vivo via Bcl-2 and caspase-3. Phytomedicine. 2018;51:214–225. 30466620 10.1016/j.phymed.2018.10.004

[9] Guo S, Chen Y, Pang C, et al. Matrine is a novel inhibitor of the TMEM16A chloride channel with anti lung adenocarcinoma effects. J Cell Physiol. 2019;234(6):8698–8708. 30370542 10.1002/jcp.27529

[10] Teh YM, Mualif SA, Lim SK. A comprehensive insight into autophagy and its potential signaling pathways as a therapeutic target in podocyte injury. Int J Biochem Cell Biol. 2021 Dec;30:106153. 10.1016/j.biocel.2021.106153 34974186

[11] Chen J, Guo Q, Chen Q, et al. Interleukin 10 inhibits oxidative stress-induced autophagosome formation in hepatic stellate cells by activating the mTOR-STAT3 pathway. Exp Cell Res. 2021 Dec;30:113001. 10.1016/j.yexcr.2021.113001 34973945

[12] Wei C, Liu X, Wang Q, et al. Identification of hypoxia signature to assess the tumor immune microenvironment and predict prognosis in patients with ovarian cancer. Int J Endocrinol. 2021 Dec 14;2021:4156187. 34950205 10.1155/2021/4156187 PMC8692015

[13] Hersant J, Ramondou P, Douillet D, et al. Comparison between conventional duplex ultrasonography and the dual-gate Doppler mode for hemodynamic measurements of the carotid arteries. Ultrasonography. 2021 Nov 10. DOI:10.14366/usg.21175 PMC8942739 34974673

[14] Han Y, Yu C, Yu Y. Astragalus polysaccharide alleviates alveolar bone destruction by regulating local osteoclastogenesis during periodontitis. J Appl Biomed. 2021 May;19(2):97–104. 34907709 10.32725/jab.2021.010

[15] Fujii SI, Yamasaki S, Hanada K, et al. Cancer immunotherapy using artificial adjuvant vector cells to deliver NY-ESO-1 antigen to dendritic cells in situ. Cancer Sci. 2021 Dec 31. DOI:10.1111/cas.15259 PMC8898705 34971473

[16] Matsoukas J, Deraos G, Kelaidonis K, et al. Myelin peptide-mannan conjugate multiple sclerosis vaccines: conjugation efficacy and stability of vaccine ingredient. Vaccines (Basel). 2021 Dec 8;9(12):1456. 34960201 10.3390/vaccines9121456 PMC8708491

[17] Young ID, Nepogodiev SA, Black IM, et al. Lipopolysaccharide associated with β-2,6 fructan mediates TLR4-dependent immunomodulatory activity in vitro. Carbohydr Polym. 2022 Feb 1;277:118606. 34893207 10.1016/j.carbpol.2021.118606

[18] Zhou YJ, Guo YJ, Yang XL, et al. Anti-cervical cancer role of matrine, oxymatrine and Sophora flavescens alkaloid gels and its mechanism. J Cancer. 2018;9(8):1357–1364. 29721044 10.7150/jca.22427 PMC5929079

[19] Singh S, Almuhanna Y, Alshahrani MY, et al. Carbohydrates from *Pseudomonas aeruginosa* biofilms interact with immune C-type lectins and interfere with their receptor function. NPJ Biofilms Microbiomes. 2021 Dec 8;7(1):87. 34880222 10.1038/s41522-021-00257-w PMC8655052

[20] Choi E, Yang J, Ji GE, et al. The effect of probiotic supplementation on systemic inflammation in dialysis patients. Kidney Res Clin Pract. 2021 Nov 18;41(1):89–101. 34974660 10.23876/j.krcp.21.014 PMC8816411

[21] Thiago AP, Mariana PP, Aline AO, et al. Human dendritic cells: their heterogeneity and clinical application potential in cancer immunotherapy. Front Immunol. 2019;9:3176. 30719026 10.3389/fimmu.2018.03176 PMC6348254

[22] Wang H, Chen H, Liu S, et al. Costimulation of γδTCR and TLR7/8 promotes Vδ2 T-cell antitumor activity by modulating mTOR pathway and APC function. J Immunother Cancer. 2021 Dec;9(12):e003339. 34937742 10.1136/jitc-2021-003339 PMC8705233

[23] Martín-Sánchez A, González-Pardo H, Alegre-Zurano L, et al. Early-life stress induces emotional and molecular alterations in female mice that are partially reversed by cannabidiol. Prog Neuropsychopharmacol Biol Psychiatry. 2021 Dec;29:110508. 10.1016/j.pnpbp.2021.110508 34973413

[24] Faitot F, Artzner T, Michard B, et al., Study Group OBOTSLTS. Immunosuppression in patients with grade 3 acute-on-chronic liver failure at transplantation: a practice analysis study. Clin Transplant. 2022 Jan;2:e14580. 10.1111/ctr.14580 34974638

[25] Tang G, Li S, Zhang C, et al. Clinical efficacies, underlying mechanisms and molecular targets of Chinese medicines for diabetic nephropathy treatment and management. Acta Pharm Sin B. 2021 Sep;11(9):2749–2767. 34589395 10.1016/j.apsb.2020.12.020 PMC8463270

[26] Aboulgheit A, Karbasiafshar C, Sabra M, et al. Extracellular vesicles improve diastolic function and substructure in normal and high-fat diet models of chronic myocardial ischemia. J Thorac Cardiovasc Surg. 2021 Oct 13;S0022-5223(21):01420–3. 10.1016/j.jtcvs.2021.07.062 PMC9005578 34756431

[27] Lynn J, Park M, Ogunwale C, et al. A tale of two diseases: Exploring mechanisms linking diabetes mellitus with alzheimer’s disease. J Alzheimers Dis. 2021 Nov 21. DOI:10.3233/JAD-210612 34842187

[28] Zhan L, Zhang J, Zhang J, et al. LC3 and NLRC5 interaction inhibits NLRC5-mediated MHC class I antigen presentation pathway in endometrial cancer. Cancer Lett. 2021 Dec 30;S0304-3835(21):00648–0. 10.1016/j.canlet.2021.12.031 34974132

[29] Wu D, Shao K, Zhou Q, et al. Autophagy and ubiquitin-mediated proteolytic degradation of PML/rarα fusion protein in matrine-induced differentiation sensitivity recovery of ATRA-resistant APL (NB4-LR1) cells: in vitro and in vivo studies. Cell Physiol Biochem. 2018;48(6):2286–2301. 30114705 10.1159/000492646

[30] Ma K, Huang MY, Guo YX, et al. Matrine-induced autophagy counteracts cell apoptosis via the ERK signaling pathway in osteosarcoma cells. Oncol Lett. 2016;12(3):1854–1860. 27588132 10.3892/ol.2016.4848 PMC4998066

[31] Ke Y, Hu TX, Huo JF, et al. Synthesis and in vitro biological evaluation of novel derivatives of Flexicaulin A condensation with amino acid trifluoroacetate. Eur J Med Chem. 2019 Nov 15;182:111645. 31494472 10.1016/j.ejmech.2019.111645

[32] Zhang X, Ming Y, Fu X, et al. PI3K/AKT/p53 pathway inhibits infectious spleen and kidney necrosis virus infection by regulating autophagy and immune responses. Fish Shellfish Immunol. 2021 Dec 28;120:648–657. 34968710 10.1016/j.fsi.2021.12.046

[33] Zittlau KI, Terradas AL, Nalpas N, et al. Temporal analysis of protein ubiquitylation and phosphorylation during parkin-dependent mitophagy. Mol Cell Proteomics. 2021 Dec;30:100191. 10.1016/j.mcpro.2021.100191 PMC8808264 34974192

